# DPP4-inhibition reduces pro-inflammatory cytokine production by alpha-beta and gamma-delta T cells in vitro and in the biliary atresia mouse model

**DOI:** 10.1038/s41598-025-16097-z

**Published:** 2025-08-18

**Authors:** Anne-Christin Wilde, Marie Uecker, Faikah Gueler, Immo Prinz, Omid Madadi-Sanjani, Claus Petersen, Joachim F. Kuebler, Christian Klemann

**Affiliations:** 1 Department of Hepatology and Gastroenterology, Charité – Universitätsmedizin Berlin, Campus Virchow-Klinikum (CVK) and Campus Charité Mitte (CCM), Berlin, Germany; 2 Department of Pediatric Surgery, Center of Surgery, Hannover Medical School, Hannover, Germany; 3 Department of Nephrology, Hannover Medical School, Hannover, Germany; 4 Institute of Systems Immunology, University Medical Center Hamburg-Eppendorf, Hamburg, Germany; 5 Hamburg Center for Translational Immunology, University Medical Center Hamburg-Eppendorf, Hamburg, Germany; 6 Present Address: Department of Pediatric Surgery, University Medical Center Hamburg-Eppendorf, Hamburg, Germany; 7 Department of Pediatric Surgery and Pediatric Urology, Hospital Bremen-Mitte, Bremen, Germany; 8 Department of Pediatric Immunology, Rheumatology and Infectiology, Hospital for Children and Adolescents, University Hospital Leipzig, Leipzig, Germany; 9 Department of Transplant Surgery, University Medical Center Hamburg-Eppendorf, Hamburg, Germany

**Keywords:** Biliary atresia, DPP4, CD26, T cells, IL-17, IFN-γ, Gamma delta T cells, Cholestasis, Gammadelta T cells

## Abstract

Etiology and pathogenesis of biliary atresia (BA) remain elusive. Evidence points to a T cell mediated autoimmune response contributing to the disease by driving the ongoing hepatic inflammation. CD26, also known as dipeptidylpeptidase 4 (DPP4), is an immunoregulatory protein also expressed on T-lymphocytes. We aimed to investigate the potential of pharmacological DPP4 inhibition on cytokine production of T cells and potential therapeutic benefits in experimental BA. We analyzed the expression and regulation of CD26/DPP4 on αβ and γδ T cells in mice suffering from rotavirus-induced BA. Enzymatic DPP4 activity in murine and human serum was examined. In cell cultures, lymphocytes were incubated with the DPP4-inhibitor Sitagliptin to study the effects of DPP4 inhibition on cytokine production. Clinical effects were assessed by intraperitoneal Sitagliptin injection of mice suffering from BA. Analyses included flow cytometry, qPCR, serum analysis and histological examinations. In mice suffering from BA, CD26/DPP4 was strongly expressed and upregulated on αβ and to an even greater extent on γδ T cells compared to healthy controls. DPP4-inhibition led to a dose-dependent suppression of the pro-inflammatory cytokines IL-17 and IFN-γ produced by Th1, Th17 and γδ T cells. Therapeutic administration of Sitagliptin in experimental BA led to reduced serum levels of GOT and Bilirubin as well as decreased hepatic infiltration with F4/80^+^ macrophages but had no effect on overall survival. In humans, serum DPP4-activity was upregulated in infants suffering from BA compared to healthy children. To our knowledge, this is the first time an upregulation of CD26 expression has been demonstrated for γδ T cells in the setting of an autoimmune inflammatory response. Mechanistically, we could demonstrate that DPP4/CD26 is upregulated on T cells in experimental BA and that pharmacological inhibition decreased their pro-inflammatory potential. However, this translated to only mild clinical benefits in the mouse model. Thus, although the protein appears to play a role in BA, further research is needed to elucidate the potential to serve as a therapeutic target.

**Supplementary Information:**

The online version contains supplementary material available at 10.1038/s41598-025-16097-z.

## Introduction

Biliary atresia (BA) is a rare cholestatic liver disease of unknown etiology that affects infants with an estimated incidence of 1:15,000–1:20,000 in the Western world^1,2^. A Kasai hepatoportoenterostomy (KPE) performed during the first weeks of life aims to restore bile flow and delay liver damage^3^. While current treatment strategies allow survival to adolescence of up to 90% of patients, the majority require a liver transplant during childhood due to terminal liver failure, making BA the most common indication for pediatric liver transplantation^3,4^. Despite extensive research efforts, BA continues to represent a relevant clinical problem with no causative treatment available.

The etiology and pathogenesis of BA remain poorly understood. The fact that patients commonly present at symptomatic stages of the disease complicates the search for a trigger, as research is limited to retrospective evaluations. Numerous factors have been discussed as possible initiators of the obliteration of bile ducts in affected patients, ranging from external toxins and genetic factors to viral or inflammatory agents^5^.

There is general agreement that a hepatobiliary (auto-)inflammatory reaction plays a critical role in the induction and progression of BA by mediating injury to the biliary epithelium and participating in the ongoing hepatic inflammation^4^. Histological and immunological analyses of liver samples have identified various immunological players (e.g., natural killer cells, macrophages, T cells, B cells) to contribute to the inflammatory environment found in the livers of affected infants^6,7^. Several studies using transcriptomic and immunohistochemical approaches have shown increased hepatic infiltration of effector CD4⁺ T cells—including Th1, Th2, and Th17 subsets—as well as a skewed Th17/Treg ratio in BA patients compared to healthy controls^8–11^. These alterations are associated with enhanced production of pro-inflammatory cytokines such as IL-17, IFN-γ, and TNF-α. In parallel, transcriptomic deconvolution and single-cell RNA-sequencing analyses have revealed expansion of cytotoxic T and NKT cells, alongside a reduction in CX3CR1⁺ effector T and NK cell subsets in BA livers^12,13^. Furthermore, recent evidence suggests that specific regulatory T cell populations, particularly ICOS⁻ Tregs, may be depleted in patients with poor outcomes, underscoring the importance of T cell balance in disease progression^8^.

Our group has previously identified that the main producers of IL-17 in experimental biliary atresia are γδ T cells. The unique population of γδ T cells plays a vital role in the neonatal organism as they bridge between the innate and adaptive immune systems^14,15^. Functionally, IL-17 was found to be upregulated in humans and mice suffering from BA, with its suppression leading to a decrease in hepatic inflammation^15,16^.

CD26/DPP4 is a ubiquitously expressed cell-surface protein with a specific enzymatic activity dipeptidyl peptidase 4 (DPP4), which is also found in serum in its soluble form^17,18^. CD26/DPP4 exhibits various immunoregulatory functions, including the modulation of chemotaxis and the ability to directly co-stimulate T cell activity. Disease-dependent regulation of CD26 expression and activity has been shown for various inflammatory and autoimmune conditions, as well as cancer and liver disease^19,20^.

The inhibition of CD26/DPP4 leads to a shift in immune response by downregulation of Th1 cell activity with subsequent suppression of their pro-inflammatory cytokine production, such as IFN-γ, TNF-α, IL-1, and IL-2 production while simultaneously upregulating Th2 activity and cytokine production^21,22^. Beneficial effects of in vivo DPP4 inhibition have been shown in experimental models of both autoimmune and liver diseases^23–25^.

In clinical care, CD26/DPP4 inhibitors are most commonly known for their use in patients with type 2 diabetes, where pharmacological inhibition leads to increased endogenous secretion of insulin through inhibition of the de-activation of glucagon-like peptide-1 and gastroinhibitory protein^18^. During the Covid-19 pandemic, a reduced mortality was shown for diabetic patients under treatment with Sitagliptin suffering from Covid-19 infection, compared to diabetic patients with different therapeutic regiments, underlining the potential of CD26/DPP4 inhibition regarding immune modulation^26^.

Considering the pivotal role of T cells in biliary atresia and the established functions of DPP4/CD26 in immune regulation and autoimmune diseases, we hypothesized that DPP4/CD26 contributes significantly to BA pathogenesis and may represent a promising immunotherapeutic target by modulating the dysregulated T cell responses characteristic of this condition, consistent with recently discussed immunotherapeutic approaches for pediatric liver diseases^7,27^. Therefore, we assessed whether CD26/DPP4 is regulated on T cells in experimental BA and extended our focus not only on αβ T helper cells but also on γδ T cell populations. While a CD26/DPP4 upregulation on αβ T cells has been shown for various autoimmune diseases, data of the expression on γδ T cells is very limited^28^. In order to evaluate the potential of DPP4 as a therapeutic target in BA, we further aimed to investigate the enzymatic DPP4 activity in infants with BA and DPP4 activity regulation in experimental BA by studying the effect of DPP4 inhibition on cytokine production and the clinical course of experimental BA.

## Methods

### Human samples

All research was conducted in compliance with the Declaration of Helsinki and in accordance with relevant guidelines and regulations. The study protocol was approved by the local ethics committee (permit numbers #41/2000 and #9270_BO_K_2020). Written informed consent was obtained from each patient’s legal guardian. Peripheral blood samples were collected from 34 infants at the time of Kasai portoenterostomy. Fourteen infants without liver disease undergoing routine surgical procedures (e.g. inguinal hernia repair, thyroglossal duct cyst resection, pyeloplasties) were used as controls.

### Animals, rhesus rotavirus, and in vivo experiments

All procedures were approved by the local animal welfare committee (permit number #42502-04-12/0785, #16/2164, § 4 2014/53). All methods were conducted in accordance with relevant guidelines and regulations, and are reported in full compliance with the ARRIVE guidelines. Adult Balb/cAnNCrl mice were obtained from Charles River Laboratories (Sulzfeld, Germany). Adult mice of the transgene line C.B6-Tcrd-Ctm1Mala strain were used as previously described to breed TCRδ H2B eGFP reporter mice which allow for distinct sorting of untouched γδ T cells by flow cytometry as they exhibit intrinsic green fluorescence signal in γδ T cells^29^. Mice were bred by our laboratory at the central animal facility under specific pathogen-free conditions. All animals were kept in pathogen-free laminar-flow cages and subjected to a 12 h dark-light cycle with food and water *ad libitum*. Mice for in vitro experiments were kept in barrier-maintained mouse colonies and fed with sterilized animal feed.

Rhesus rotavirus (RRV) strain MMU18006 was acquired from American Type Culture Collection (Manassas, USA) and grown in MA104 cells (ATCC, Manassas, USA). Virus titration was performed according to Lindenbach et al.^30^. For the induction of BA, mice were injected intraperitoneally within 24 h of life with a phosphate-buffered saline (PBS)/RRV solution containing 230 focus-forming units of RRV in 50 µl.

For experiments testing DPP4 inhibition BA mice were subjected to intraperitoneal injections of Sitagliptin (Selleck Chemicals, Houston, USA) in PBS or PBS only (control group). Sitagliptin injections were started 24 h after birth and given once daily until the end of the experiment. Dosage indications for Sitagliptin are limited to adult rats (10-30 mg/kg/d), adult mice (10–100 mg/kg/d) and neonatal rats (1.5 mg/kg/d–2.8 g/kg/month), no data for neonatal mice exists. Based on these dosages, we calculated a dosage of 33 µg Sitagliptin per injection (= 20 mg/kg/d) for our experiments, taking into account the increased metabolism of neonates as well as a three to four-fold increase in body weight over the course of the experiment. In order to rule out toxicity, healthy neonates were subjected to intraperitoneal injection with the stated dosage of Sitagliptin (33 µg Sitagliptin in 30 µl PBS) or 30 µl PBS for controls. The experimenter performing the injections was blinded to the contents of the injected solution.

Mice were monitored daily. Clinical parameters (jaundice, oily fur, weight; Table 1) as well as survival was assessed. Animals were sacrificed by decapitation or cervical dislocation. Blood was collected and preserved for serum analysis. Explanted livers were either directly processed for FACS analysis, stored in 4% isopentan for histological analysis or frozen at – 80 °C in RNA later buffer for qPCR analysis.

**Table 1:** Observational score for clinical evaluation of mice.

Score	0	5	10	15
Symptoms				
Jaundice	No	No	Yes	Death during observational period
Oily fur	No	In parts	Yes	
Weight	Increase	Stagnation	Decrease	

### Cell isolation and purification

Leukocytes were isolated from liver, spleen, and lymph nodes from 9 to 15 weeks old adult Balb/cAnNCrl or TCRδ H2B eGFP reporter mice. Liver infiltrating leukocytes from mice with BA were extracted as previously described by tissue homogenization, density gradient centrifugation, magnetic activated cell sorting (MACS), and fluorescent activated cell sorting (FACS)^15,16^. Quality control by flow cytometry revealed a purity of > 99% of αβ and γδ isolates (not shown). γδ T cells were collected using the TCRδ H2B eGFP reporter mouse with intrinsically fluorescent γδ T cells that allow for FACS-sorting of non-activated γδ T cells^29^.

For toxicity-testing of DPP4-inhibitors on T helper cell subpopulations, naïve T cells were isolated with the Naive CD4^+^ T Cell Isolation Kit, mouse (Miltenyi Biotech, Bergisch Gladbach, Germany) according to the manufacturer’s protocol and then differentiated to Th1 and Th17 cells as described previously^31^.

### Cell cultures

Conditions for cell culture experiments were set as previously described^15,16^. For experiments using DPP4-inhibitor Sitagliptin, a masterstock (100 µM) in Dimethyl sulfoxide (DMSO) was produced, aliquoted and stored at – 80 °C. Solutions were diluted with RPMI medium to different concentrations as needed for experiments.

#### Estimation of DPP4 dosage

2 × 10^5^ cells per well of isolated leukocytes from healthy adult mice were incubated in four groups (no stimulation, stimulation with anti-CD3 only, stimulation with IL-23 only, stimulation with anti-CD3 and IL-23) with increasing concentrations (0 µM, 5 µM, 25 µM, 50 µM, 100 µM, 250 µM, 350 µM, 500 µM) of the DPP4-inhibitor Sitagliptin for 72 h. Numbers of viable cells were then estimated with the trypan blue dye exclusion test. While small dosages (5 µM) led to a minor but statistically not significant reduction in viable cells, a significant reduction was only found after incubation with dosages of 700 µM or higher.

#### DPP4 inhibition of Th1 cells, Th17 cells and αβ T cells

Naive CD4^+^ T cells were differentiated into Th1 cells and Th17 cells as previously described^15^. Cells were then co-incubated with either DPP4 inhibitor or PBS for controls. Cell culture supernatants were tested for concentrations of IFN-γ and IL-17 as previously described^16^.

MACS-isolated αβ T cells from adult mice were divided into two groups, both of which were mixed with anti-CD28 (1 µg/ml) (eBioscience GmbH, Frankfurt, Germany) and the second group additionally with IL-23 (10 ng/ml). 2 × 10^5^ cells per well of each solution were exposed to different concentrations of Sitagliptin (0 µM, 5 µM, 25 µM, 100 µM, 500 µM in RPMI), incubated for 72 h and supernatants then stored for ELISA analysis.

#### DPP4 inhibition of intrahepatic leukocytes from BA mice

Isolated murine intrahepatic leukocytes (IHL) from mice with BA were divided into two groups, both of which were mixed with anti-CD3 (0.5 µg/ml) (eBioscience GmbH, Frankfurt, Germany) and the second group additionally with IL-23 (10 ng/ml). Sitagliptin was added in increasing concentrations (0 µM, 25 µM, 100 µM, 250 µM, 500 µM). After incubation for 24 h, supernatants were stored for ELISA analysis.

### Analysis

#### Flow cytometry

FACS staining was performed as previously described^15^. Surface staining was performed for T cell phenotyping using the following antibodies TCRyd (1:100, conjugate FITC, eBioscience), CD26 (1:50, conjugate PE, BioLegend), CD3e (1:200, conjugate PerCP-Cy5.5, eBioscience), CD8a (1:200, conjugate PE-Cy7, eBioscience), TCRb (1:600, conjugate APC, eBioscience), CD4 (1:600, eF780, eBioscience), CD45 (1:600, conjugate eF450, eBioscience) as well as an FcR-block (1:100). After incubation with PFA solution cells were flushed and mixed with 50–300 µl FACS buffer depending on the number of cells. Samples were acquired on a FACS Canto II flow cytometer (BD) and analyzed with FACS Diva software (BD) and KALUZA software (Beckman Coulter, Brea, USA).

#### Cytokine assays

Cytokine concentrations (IFN-γ and IL-17) in cell culture supernatants were quantified using DuoSet ELISA Development Kits (R&D Systems, Minneapolis, USA) following the manufacturer’s instructions. Briefly, 96-well plates were coated with capture antibodies, blocked, and incubated with standards or appropriately diluted samples. After incubation with biotinylated detection antibodies and streptavidin-HRP, a colorimetric reaction was induced using substrate solution and stopped with a stop buffer. Absorbance was measured at 450 nm with wavelength correction at 540–570 nm using a Glomax microplate reader (Promega).

#### Quantification of DPP4 activity

Enzymatic activity of DPP4 in cell cultures as well as animal serum was assessed using DPP4-Glo™ Protease Assay (Promega GmbH, Mannheim, Germany) according to the manufacturer’s protocol. The concentration of DPP4 in the serum of neonatal mice was initially not known. Based on amounts in human serum (0.3 µl/ml), animal serums were diluted with Tris buffer (1%, 0.1%, 0.01%, 0.001%) to match the quantitative detection abilities of the assay (0.3 pg/ml−1 ng/ml). 20 µl of undiluted cell culture was mixed with 20 µl of the DPP4-Glo™ reagent. A mix of 20 µl cell culture and 20 µl PBS was used as a negative control. PBS mixed with DPP4-Glo™ and recombinant DPP4 served as a positive control. After 30 min of incubation, luminescence was measured photometrically with a MicroplateReader (GloMax-Multi + Microplate Reader, Promega, Walldorf, Germany).

#### RNA extraction and quantitative PCR

RNA was isolated from frozen livers and transcribed to cDNA as previously described using QuantiTect Primer (Quiagen)^15,16^.

#### Serum analysis

Serum analysis (GOT, GPT, Bilirubin) was performed using an AU 400 Olympus Analyzer (Olympus, Tokyo, Japan) as previously described^32^.

#### Histology

Histological examinations were performed as previously described^15^. Liver sections were processed as previously described with antibodies rat anti-mouse GR-1, rat anti-mouse F4/80 and anti-CD3 (Clone: 145-2C11) (AbD Serotec, Oxford, UK) and stained with fluorescence dye DAPI^16,32^.

### Statistics

Statistical analysis was performed using GraphPad Prism software version 9.0 (GraphPad Software, San Diego, USA). Data were assessed for normality using the Shapiro-Wilk test prior to analysis. Two groups were compared using the unpaired t-test. ANOVA with Sidak’s multiple comparison test was used to compare more than two groups. Survival analysis was performed with log-rank tests of Kaplan-Meier curves. *P* < 0.5 was considered statistically significant and is indicated with * in the figures (*p* < 0.01 = **, *p* < 0.001 = ***).

## Results

### CD26/DPP4 is expressed and upregulated on αβ and γδ T cells in experimental BA

It has previously been demonstrated that Th1 cells and IL-17 producing γδ T cell populations contribute to the development of BA^15,33^. Besides its enzymatic function, CD26/DPP4 has been shown to have a direct co-stimulatory effect on T cells^18^. We therefore started off our experiments by analyzing bulk intrahepatic leukocytes (IHL) over the disease progression of BA to determine whether CD26/DPP4 is expressed and regulated in experimental BA. Flow cytometric analysis of intrahepatic T cells of healthy mice and mice with BA showed a continuous and significant increase of the fraction and absolute numbers of CD3^+^ T-cells in the livers of affected animals (Suppl. Fig. 1A + C), with a markedly increased fraction of CD26 expressing T cells as the disease progressed (Suppl. Fig. 1B + C). In mice suffering from BA, 91% of intrahepatic T cells tested positive for CD26 on day 14 as opposed to 69% of intrahepatic T cells from healthy controls (Suppl. Fig. 1C).

We next assessed which T cell subsets are to be attributed for the increased CD26 expression by analyzing T cell subpopulations. CD4⁺ and CD8⁺ αβ T cells were analyzed separately for CD26 expression due to their distinct roles in adaptive immunity and potential differences in activation status. γδ T cells were stratified into CD8⁺ and double-negative (DN) subsets to explore subset-specific expression of CD26, as these populations differ in tissue homing and effector function.

**Fig. 1:**
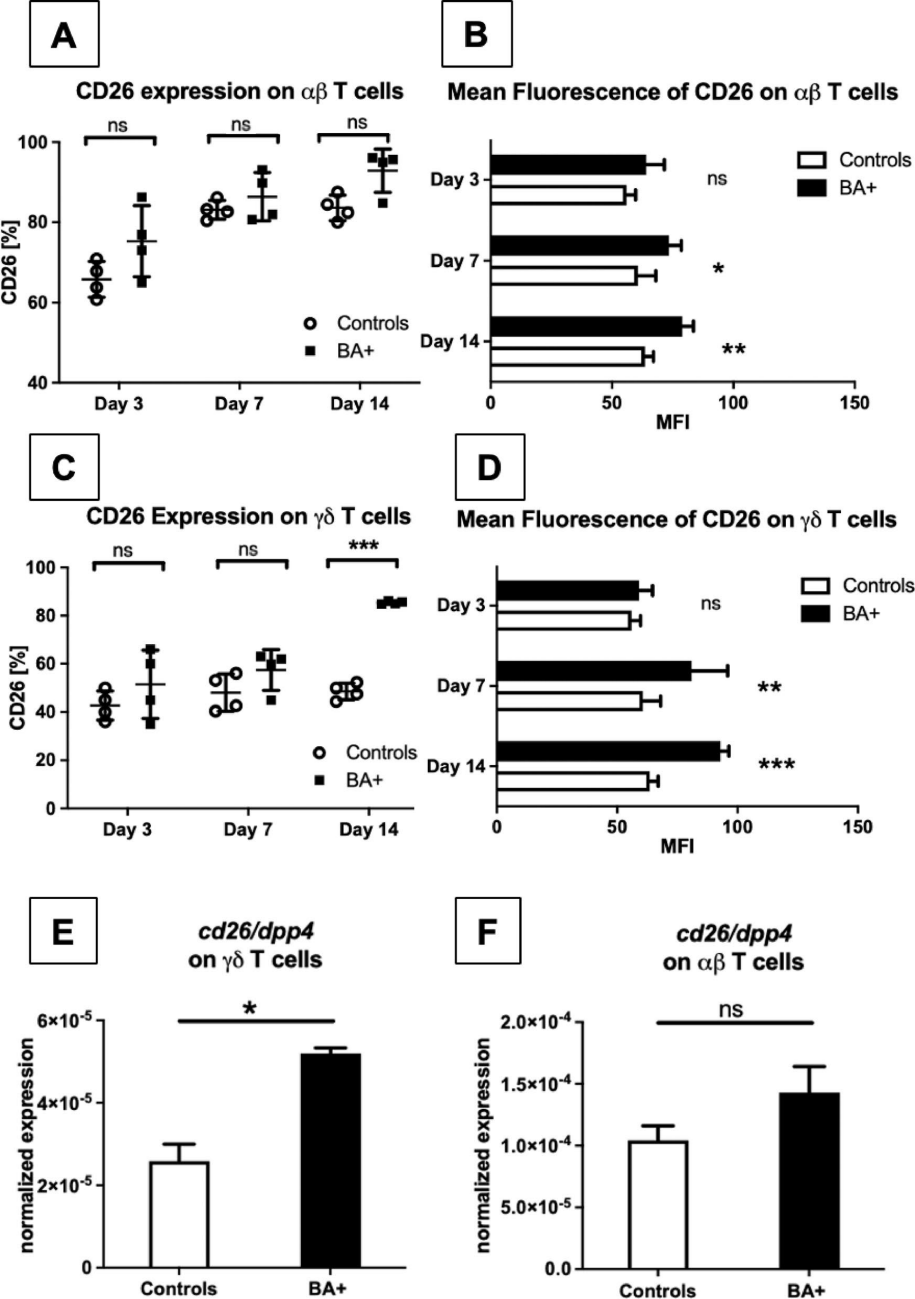
CD26 is expressed and upregulated on αβ T cells and to a higher extent on γδ T cells in experimental BA. **A/C**: *n* = 4 pools of intrahepatic leukocytes (consisting of 2–3 animals each) of mice with BA and healthy controls were analyzed on day 3, 7, and 14 by flow cytometry for CD26 expression on αβ T cells (**A**) and γδ T cells (**C**). Flow cytometry was performed using a panel targeting murine surface markers, including TCRγδ, CD26, CD3e, CD8a, TCRβ, CD4, and CD45. MFI was assessed on the same day with the same flowmetric settings in αβ T cells (**B**) and γδ T cells (**D**). Additionally, *n* = 3 pools (consisting of 5–10 animals each) of highly purified, FACS sorted CD45^+^CD3^+^TCRγδ^+^ (**E**) and CD45^+^CD3^+^TCRαβ^+^CD4^+^ (**F**) were analysed by quantitative reverse transcription PCR to measure the cd26/dpp4 expression relative to glyceraldehyde-3-phosphate dehydrogenase (GAPDH) as a housekeeping gene. Data represent three independent experiments. Groups were compared by unpaired t-test. ANOVA was performed for comparison of individual groups (healthy controls/BA) over the time period, showing a significant increase in CD26 expression in αβ T cells of healthy controls (*p* < 0.0001) and BA mice (*p* < 0.05) as well as γδ T cells in BA mice over time (*p* < 0.01). ANOVA analysis of CD26 MFI revealed a significant increase in both αβ T cells and γδ T cells in mice with BA (*p* < 0.05 and *p* < 0.01 respectively) while healthy mice showed no increase of CD26 MFI over time. *p < 0.05; **p < 0.01; ***p < 0.001; ns = not significant.

αβ T cells showed already high basal expression rates of about 70% CD26 in both healthy and BA-infected mice (Fig. 1A). No significant increase in the number of CD26^+^ cells was found after induction of BA compared to healthy controls (Fig. 1A), but the amount of CD26 expression on each αβ T cell measured by CD26 Mean Fluorescence Intensity (MFI) showed a significant increase over the course of the disease (Fig. 1B). Both healthy and BA mice showed a significant increase of CD26 expression on αβ T cells within their group over time (*p* < 0.0001 healthy controls, *p* < 0.05 BA mice, not shown). No differences were found in expression levels of CD26 between CD4^+^ or CD8^+^ αβ T cell subpopulations (data not shown).

Our group has previously shown that IL-17 producing γδ T cells contribute to the development of BA in the mouse model^15^therefore we investigated these cells for their CD26 expression. Our data demonstrated a lower basal rate of CD26 expression of about 40% in γδ T cells as opposed to about 70% in the αβ T cell population in the livers of healthy animals (Fig. 1A + C). While CD26 expression over time did not show a substantial increase in the group of healthy controls, a significant elevation was found in γδ T cells of BA mice with significant increase in CD26 expression compared to healthy controls by day 14 (*p* < 0.001, Fig. 1C).

No significant difference in CD26 expression on γδ T cells between BA mice and healthy controls was found in the early phases of BA development on day 3 and day 7 of life (Fig. 1C). Interestingly, in contrast to the moderate increase in CD26 expression found in αβ T cells over the observed time period, CD26 expression in γδ T cells doubled up to about 80% of γδ T cells expressing CD26 in the late course of the disease (Fig. 1C). CD26 Mean Fluorescence Intensity (MFI) of γδ T cells, as an indicator of the amount of CD26 molecules expressed on the surface, was also significantly elevated in BA mice compared to healthy controls on day 7 and day 14 (Fig. 1D).

To assess the transcriptional activity, we additionally performed qPCR analysis of intrahepatic T cells isolated from the livers of mice with BA and healthy controls. After flow cytometric separation of αβ and γδ T cells the expression level of *cd26/dpp4* mRNA was measured, confirming that basal expression of CD26/DPP4 on αβ T cells is higher compared to γδ T cells, in which induction of BA led to a significant upregulation (Fig. 1E + F).

Taken together, our results demonstrate that CD26/DPP4 is expressed on most αβ T cells under baseline conditions and increases after induction of BA as indicated by rising MFI, especially in the later stages of the disease. Baseline CD26 expression is much lower on γδ T cells but the number and amount of CD26 expression greatly increase over the course of BA in this subpopulation.

### DPP4 activity in serum is increased in BA

**Fig. 2:**
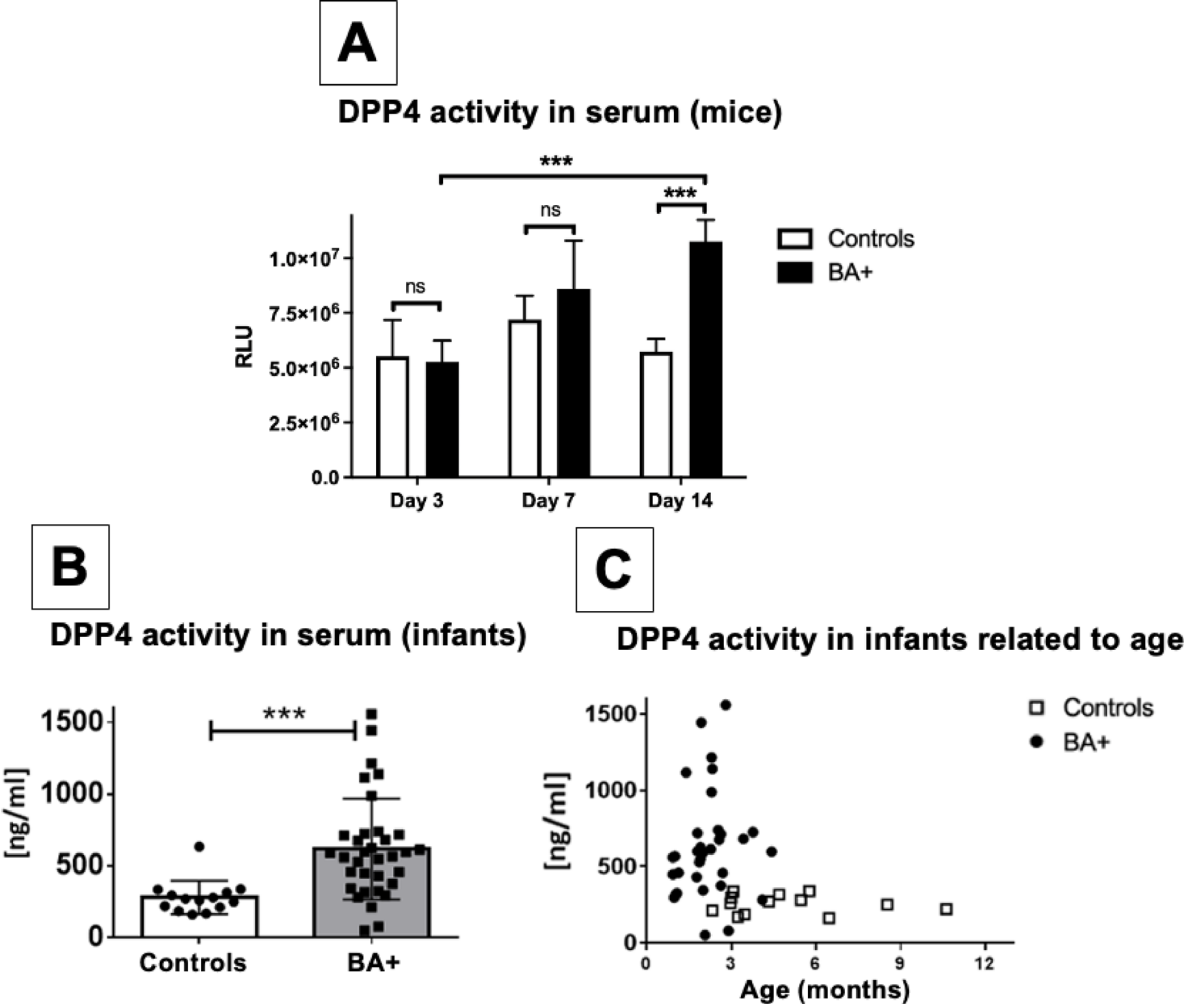
DPP4 activity in serum is increased in mice and infants with BA. **A**: Serum of mice with and without BA was taken from 6–10 individual mice per group on DOL 3, 7, and 14. DPP4 activity was assessed using a DPP4 bioluminescence assay by measuring relative light units (RLU). A standard curve for conversion of RLU into ng/ml was generated using human recombinant DPP4. **B/C**: Serum was taken from infants with BA (*n* = 34) at the time of kasai portoenterostomy and infants without liver disease (*n* = 14) during routine surgery and analyzed using a DPP4 bioluminescence assay. Error bars represent the standard deviation. Groups were compared using unpaired t-test. *** *p* < 0.001; ns = not significant.

Besides its role as a co-stimulatory T cell receptor, CD26 has an enzymatic activity, referred to as DPP4. Therefore, the sera of mice suffering from BA and healthy controls were analyzed using a bioluminescence assay to determine this enzymatic activity from soluble CD26. In animals suffering from BA, enzymatic DPP4 activity significantly increased during the course of the disease and was twice as high as in healthy controls by day 14 (Fig. 2A).

In a translational approach, we compared the DPP4 activity in serum from 34 infants with confirmed BA, obtained at the time of KPE, with the DPP4 activity in serum from 14 immunologically healthy infants who underwent routine surgery (e.g. hernia repair). DPP4 activity in serum was significantly higher in infants suffering from BA (619 ± 60.3 ng/ml) compared to healthy controls (281 ± 31.3ng/ml) (*p* < 0.001) (Fig. 2B). The enzymatic activity was not related to the age at the time-point of KPE (Fig. 2C).

### DPP4 inhibition in BA leads to reduced enzymatic DPP4 activity in vitro

As our previous experiments demonstrated an upregulation of CD26/DPP4 expression as well as enzymatic DPP4 activity in BA, we set out to investigate the effect of pharmacological DPP4 inhibition on the cytokine production of αβ and γδ T cells in vitro. We first conducted dose-finding experiments to investigate if the DPP4 inhibitor Sitagliptin sufficiently suppresses enzymatic DPP4 activity in our in vitro experimental setting. We chose Sitagliptin because it has a high specificity against DPP4 and was the first DPP4 inhibitor approved by the Food and Drug Administration (FDA) for use in type 2 diabetes in 2006 with successful use in patients since. Starting at 5 µM all concentrations of Sitagliptin caused an approximately twenty-fold reduction in DPP4 activity compared to controls (0 µM) (*p* < 0.001) with no overt toxicity (Suppl. Fig. 3). Confirmatory experiments demonstrated a dose-dependent DPP4-inhibition of Sitagliptin in αβ T cells purified from healthy mice as well as IHL isolated from mice with BA that had been restimulated in vitro (Suppl. Fig. 3).

### DPP4 inhibition reduces the number of IL-17^+^ γδ T cells and the amount of pro-inflammatory cytokines produced in vitro and ex vivo

The pro-inflammatory cytokines IL-17 and IFN-γ produced by Th1^33^ and γδ T cells^15^ are pivotal perpetuators of the progressive inflammatory destruction in the hepatobiliary system in BA. Therefore, we investigated whether DPP4 inhibition impacts their production in T cell stimulation assays in vitro.

**Fig. 3:**
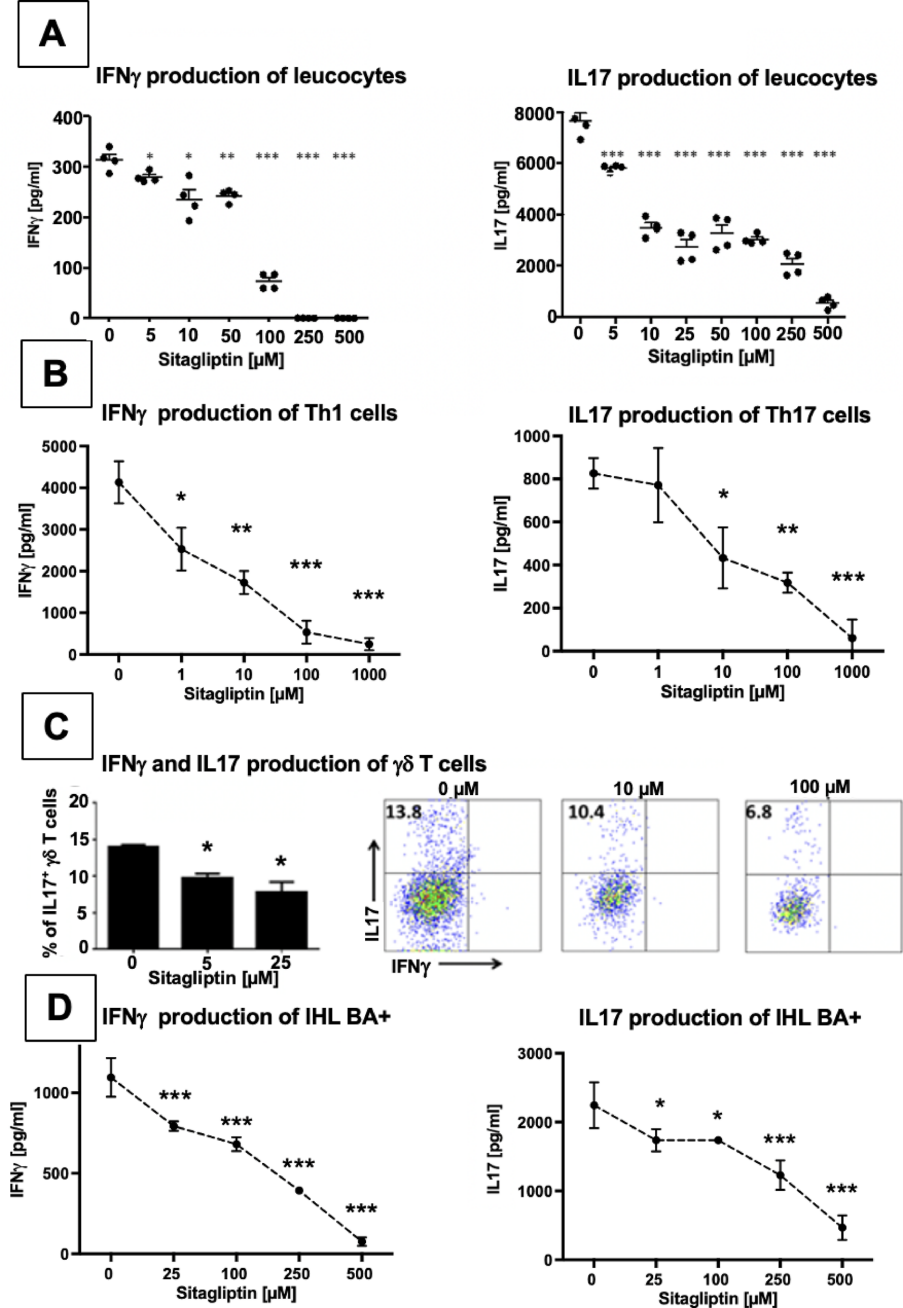
DPP4 inhibition leads to reduced production of IFN-γ and IL-17 of αβ and γδ T cells. **A**: Leukocytes were isolated from spleen and lymph nodes of 10–12 weeks old healthy mice. 2 × 10^5^ cells per well were stimulated with soluble anti-CD3-antibody and recombinant IL-23 and incubated with increasing concentrations of Sitagliptin in triplicates for 72 h. The concentrations of IFN-γ and IL-17 were measured in supernatants by ELISA (R&D Systems). **B**: 2 × 10^5^ naïve CD4^+^ T helper cells per well were stimulated with anti-CD28, anti-IL-4, IL-2, and IL-12 for differentiation into IFN-γ producing Th1 cells or with IL-6 and TGF-β for differentiation into IL-17 producing Th17 cells. 2 × 10^5^ cells per well were then incubated on an anti-CD3-antibody coated plate with increasing concentrations of Sitagliptin in triplicates for 72 h. Supernatants were analyzed for IFN-γ and IL-17 concentrations using ELISA. **C**: γδ T cells from spleen and lymph nodes of healthy TCRδ H2B eGFP reporter mice were FACS-sorted using their intrinsic fluorescence. 2 × 10^5^ cells per well were stimulated with increasing concentrations of Sitagliptin for 24 h. IL-17 and IFN-γ production was assessed by intracellular cytokine staining and flow cytometry. Representative FACS plots show IL-17 and IFN-γ expression in CD45⁺CD3⁺GFP⁺ (TCRγδ⁺) cells. **D**: Intrahepatic leucocytes were isolated from 8 to 10 mice with BA on DOL14. 2 × 10^5^ cells per well were stimulated with soluble anti-CD3-antibodies and increasing concentrations of Sitagliptin for 24 h in duplicates. IL-17 and IFN-γ concentrations in supernatants were analyzed with ELISA. Error bars represent the standard deviation. Unpaired t-test was used to compare two groups, multiple comparisons were done by ANOVA. * *p* < 0.05; **p < 0.01; ***p < 0.001; ns = not significant.

In leukocyte cultures of healthy mice, a significant reduction of both IFN-γ and IL-17 could be observed already at low concentrations of 5 µM Sitagliptin with an increasing dose-dependent effect with higher dosages up to a four-fold reduction (Fig. 3A). These cell cultures contain multiple other immune cells which might also have the ability to produce IL-17 or IFN-γ. To demonstrate a specific effect of DPP4 inhibition on cytokine production in T cells, we tested the effect of DPP4 inhibition on cytokine production in isolated T cell cultures. Naïve CD4^+^ T helper cells were first isolated from healthy mice and then differentiated into Th1 and Th17 T cells in vitro. Adding a DPP4 inhibitor to these cell cultures resulted in a significant dose-dependent reduction of cytokine production upon restimulation (Fig. 3B). In isolated γδ T cell cultures, addition of Sitagliptin led to a dose-dependent significant decrease in IL-17-producing γδ T cells to about half of their initial fraction (Fig. 3C).

Cells exposed to higher dosages of Sitagliptin did not show decreased numbers of viable cells, rendering a cytotoxic effect of high Sitagliptin concentrations unlikely (Suppl. Fig. 3A). Altogether, this suggests that the observed decrease in cytokine production is due to a suppression of inflammatory pathways, altered T cell differentiation, and/or T cell co-stimulation and is not attributable to an antiproliferative or toxic effect of high Sitagliptin dosages.

As these experiments with T cells isolated from healthy animals and artificially differentiated into effector T cells in vitro may not reflect the inflammatory conditions in vivo, we aimed to evaluate the effect of DPP4 inhibition on the cytokine production ex vivo by using pathogenic, highly differentiated effector T cells isolated from the livers of mice suffering from BA. In those, DPP4 inhibition also led to a dose-dependent, highly significant decreased production of IFN-γ and IL-17 with an about 12-fold reduction of IFN-γ and a 7-fold reduction in IL-17 concentration (Fig. 3D).

Taken together, DPP4 inhibition led to a decrease of IL-17 and IFN-γ producing T cells without an observed cytotoxic effect, suggesting a modulation of immunoregulatory pathways by DPP4 inhibition. In addition, we found a strikingly reduced amount of cytokine production both in isolated T cell cultures from healthy mice as well as in T cell cultures of in vivo differentiated intrahepatic T cells of mice suffering from BA, suggesting a distinct effect of DPP4 inhibition on cytokine production of T cells in BA.

### Treatment with sitagliptin of mice with BA lowers GOT and bilirubin

**Fig. 4:**
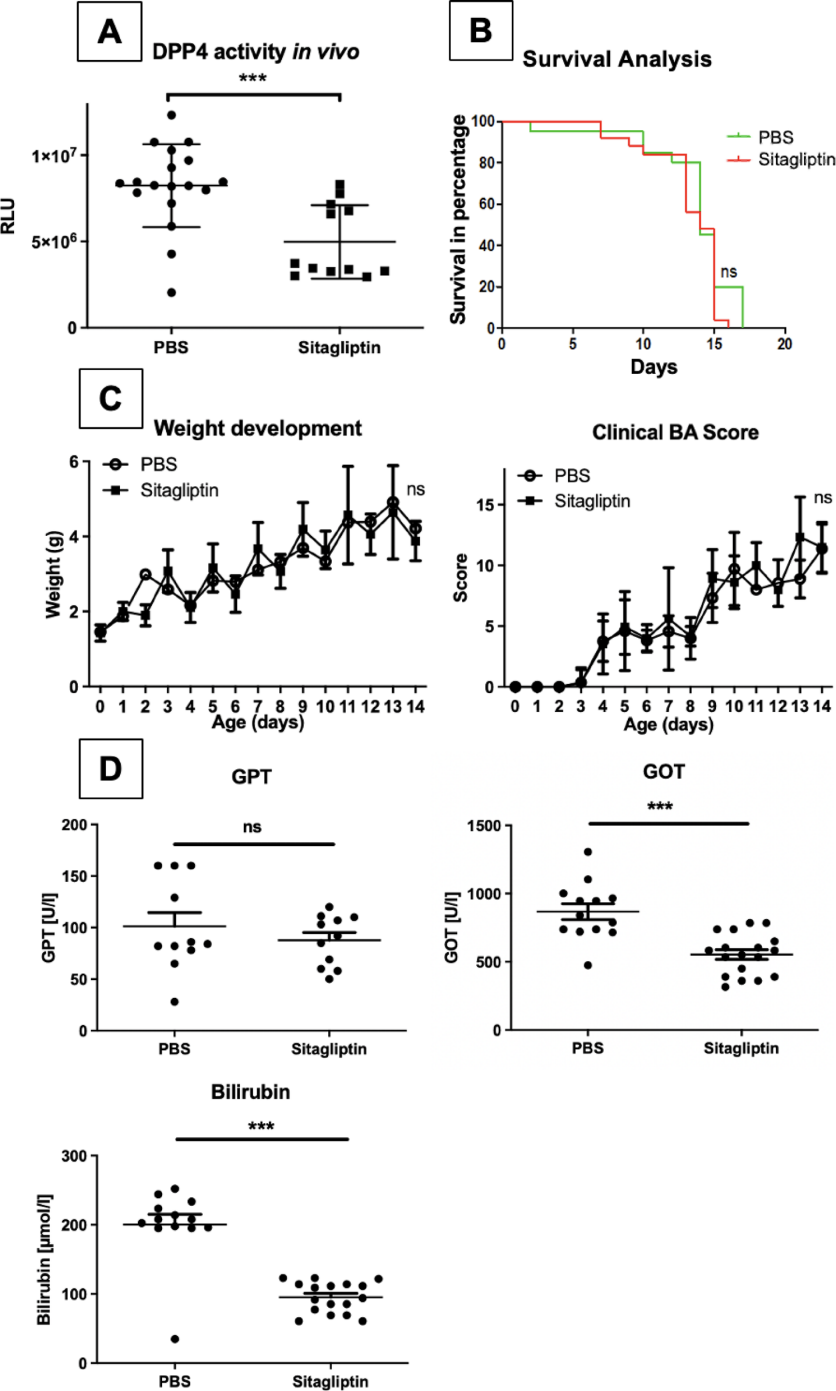
DPP4 inhibition with Sitagliptin in BA leads to mild clinical improvement in vivo. 24 h after induction of BA in balb/c mice animals received daily intraperitoneal injections with 33 µg Sitagliptin in 30 µl injection volume (approximately 20 mg/kg/day) or 30 µl PBS by a blinded investigator. **A**: Serum was taken on DOL 14 and DPP4 activity was measured by bioluminescence assay (Sitagliptin *n* = 12, PBS *n* = 18). Each data point represents a single mouse. **B/C**: Mice were monitored daily for clinical condition, activity and weight. If weight loss or decrease in activity or clinical condition was observed, mice were sacrificed with decapitation. The Kaplan-Meyer survival analysis is shown in **B** (Sitagliptin *n* = 25, PBS *n* = 21, *p* = 0.21). Weight development and clinical BA score are shown in **C** (Sitagliptin *n* = 23, PBS *n* = 16). **D**: Serum of mice taken on DOL12-14 was analyzed for concentrations of GOT, GPT, and Bilirubin. (GPT: Sitagliptin *n* = 11, PBS *n* = 11, *p* = 0.38; GOT: Sitagliptin *n* = 18, PBS *n* = 13, ***p < 0.001; Bilirubin: Sitagliptin *n* = 17, PBS *n* = 13, ***p < 0.001). Error bars represent the standard deviation. Group comparisons were performed by unpaired t-test. *** *p* < 0.001, ns = not significant.

In order to assess the clinical relevance of DPP4 inhibition, we tested its effect on the clinical course of BA in the mouse model. Analysis of serum taken at the end of the experimental course (day 12–14) showed an approximately 1,7-fold decrease of DPP4 activity in the serum of mice injected with Sitagliptin compared to the control group, which proved statistically significant (PBS Controls: 8236396 ± 2399422 RLU; Sitagliptin: 4972839 ± 2125118 RLU, *p* < 0.001) (Fig. 4A). However, while DPP4 inhibition proved effective to reduce levels of GOT (PBS Controls: 808.0 ± 251.5 U/L; Sitagliptin 553.2 ± 150.7 U/L, *p* < 0.001) and Bilirubin (PBS Controls: 186.2 ± 73.74 U/L; Sitagliptin 95.1 ± 22.26 U/L, *p* < 0.001) compared to controls (Fig. 4D), no significant impact on survival, weight development or clinical BA score was observed (Fig. 4B + C).

**Fig. 5:**
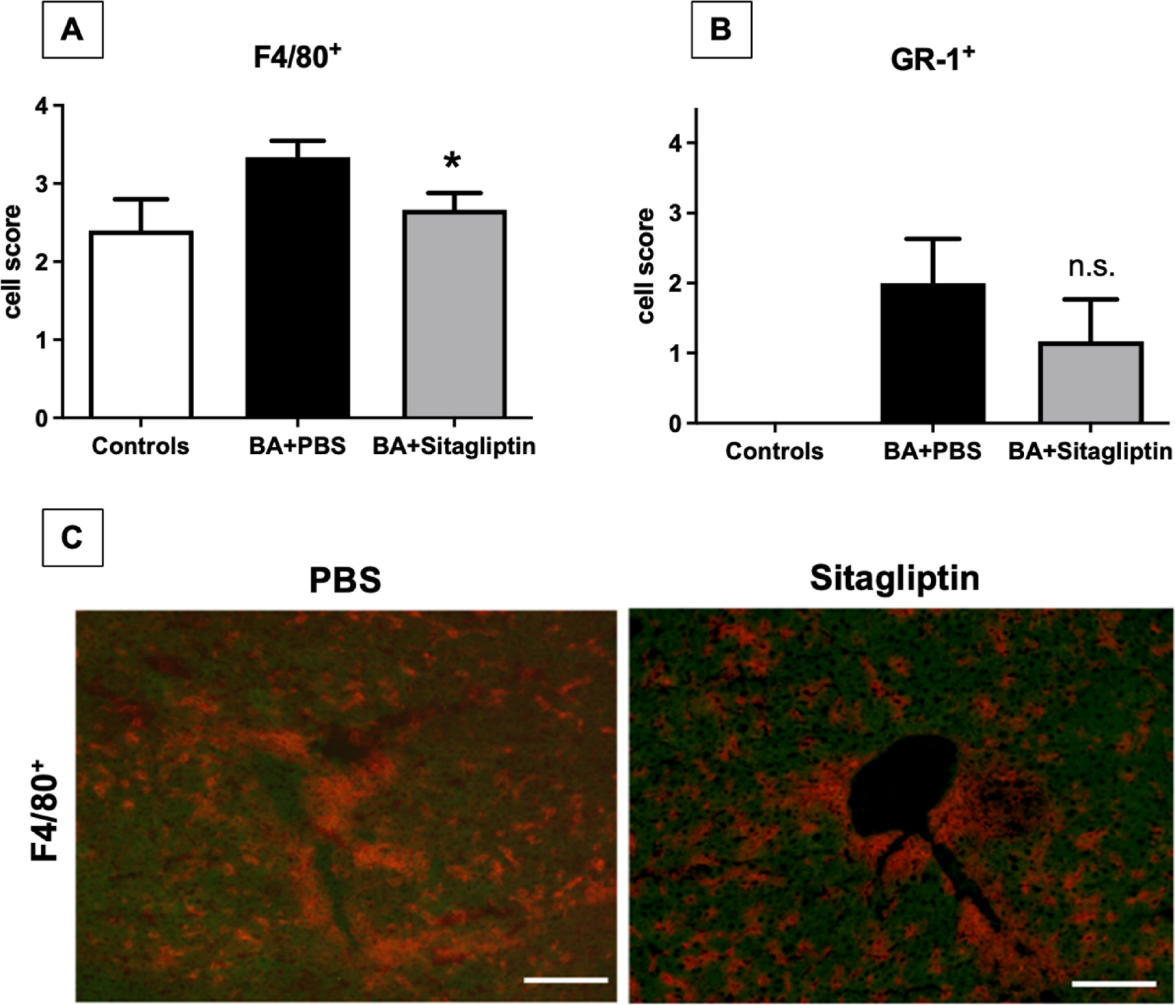
DPP4 inhibition leads to a reduction of F4/80^+^ macrophages in the liver in vivo. Liver cryosections of mice with BA treated with Sitagliptin or injected with PBS for controls were prepared for immunohistochemistry and stained with F4/80^+^- and GR-1 antibodies. **A/B**: To quantify periportal leukocyte cell infiltration 10 different frames per liver section were analyzed in 20-fold magnification of healthy control animals and mice with BA treated with either PBS or Sitagliptin. A semi-quantitative scoring system was used: score 0, no infiltrating cells per view field; 1, mild infiltration with < 10cells/view field; 2, moderate inflammation with 11–25 cells/view field; 3, severe inflammation with 26–50 cells/view field; and 4, dense infiltrates with > 51 cells/view field. ANOVA was performed and levels of significance are given as *p < 0.05; ns = not significant. **C**: Representative slides of liver tissue from mice with BA treated with PBS or Sitagliptin, showing a reduction of F4/80^+^ cells with Sitagliptin treatment. (Bar = 200 μm)

One of the striking features of BA is the periportal inflammation. Therefore, we analyzed whether DPP4 inhibition impacts periportal inflammation by investigating infiltration of immune cells, namely macrophages and granulocytes in the liver of healthy mice and mice with BA treated with PBS or Sitagliptin. Histological analysis showed a significant reduction of F4/80^+^ macrophages in BA mice treated with Sitagliptin (Fig. 5A + C), but no difference in the influx of GR-1^+^ neutrophile granulocytes was observed (Fig. 5B).

## Discussion

The exact etiology and pathogenesis of BA remain elusive, but a dysregulated immune response appears to be the major driver of disease progression. It has previously been demonstrated that experimental BA is driven by IFN-γ producing Th1 cells and IL-17 producing γδ T cells, both of which are also upregulated in human BA^7,15^. As the ubiquitously expressed ectopeptidase CD26/DPP4 is involved in numerous inflammatory and autoimmune conditions^18,34^, we aimed to investigate the expression and regulation of CD26/DPP4 in human BA as well as the effect of DPP4 inhibition on the production of the predominant cytokines IL-17 and IFN-γ in experimental BA.

### CD26/DPP4 and neonatal αβ T cells in BA

Our assessment of T cell kinetics in mice suffering from BA demonstrated a strong increase in T cell numbers in the livers of mice with BA, supporting the hypothesis of a T cell-based autoimmune response as a central player in the pathogenesis of BA (Suppl. Fig. 1). Baseline expression of CD26 on intrahepatic αβ T cells from healthy, neonatal mice were already high (Fig. 1A), supporting previous data reporting basal CD26 expression rates of up to 70% on Th cells in adult mice^18,35^. This high basal expression suggests a limited dynamic range for CD26 modulation in αβ T cells and implies that this subset may have a less prominent role in the CD26 associated immune dysregulation observed in BA. While our finding of a modest CD26 upregulation on αβ T cells during disease progression is consistent with previous studies demonstrating CD26 regulation on αβ T cells in autoimmune and inflammatory models^36,37^these results suggest that more relevant regulatory shifts in CD26 expression in BA are likely driven by other T cell subsets, such as γδ T cells.

### CD26/DPP4 and neonatal γδ T cells in BA

γδ T cells are considered essential to neonatal immune function^38^. We have previously demonstrated that γδ T cells are pivotal in disease development in BA^15^. Data on CD26 expression on γδ T cells are very scarce. Juno et al. studied CD26 expression levels on γδ T cells of patients with end-stage renal disease (ESRD) and found a significant reduction of CD26 expressing γδ T cells compared to healthy controls. They additionally found a decreased proportion of CD26^+^γδ T cells expressing chemokine receptors, suggesting a loss of costimulatory properties of γδ T cells in the setting of ESRD. Even though we found similar basal CD26 expression levels on γδ T cells in healthy controls (approx. 30%) the significant upregulation of CD26 expression on γδ T cells to about 75% after induction of BA stands in steep contrast to the results of Juno et al., which are likely to be attributed to different pathophysiologies of the underlying diseases.

Despite these differences, both studies confirm that CD26 is expressed on γδ T cells and shows disease-dependent regulation.

### Enzymatic DPP4 activity in BA

Besides the CD26 expression on cell surfaces the enzymatic DPP4 activity has also been studied in the context of autoimmune conditions, showing both up- and downregulation in different experimental disease models^39,40^, as well as human diseases^41,42^. The enzymatic DPP4 activity is primarily caused by soluble DPP4 (sCD26). The exact source of sCD26 remains unknown, but is suspected to be caused by ‘shedding’ from the surface of activated immune cells, with the enzyme staying active after separation from the membrane^43,44^. Our findings of increased serum DPP4 activity in both experimental and human biliary atresia (BA) align with previous reports demonstrating elevated DPP4 activity in the urine and serum of infants with BA^45,46^. As increased DPP4 serum activity has been found in multiple hepatological diseases, such as hepatocellular carcinoma, NASH or cirrhosis, with DPP4 showing ubiquitous expression in bile ducts and adjacent hepatocytes^17,47,48^we deem our observations to be unspecific to BA, but rather interpret the DPP4 activity as a sign of a general activation of the immune system in the setting of hepatic inflammation. Further studies incorporating larger cohorts and more comprehensive clinical data are needed to better evaluate the role of DPP4 in clinical BA. However, based on current evidence, its diagnostic and prognostic value in the disease-specific context of BA appears to be limited.

### Effect of DPP4 inhibition on inflammatory cytokine production by αβ and γδ T cells

CD26/DPP4 has been shown to influence T cell regulation and cytokine production with DPP4 inhibition leading to decreased inflammatory profiles^49,50^. In CD4^+^ αβ T cells, Pinheiro et al. reported a decrease of cell numbers as well as their production of IFN-γ and IL-17 in human lymphocytes^51^. Wang et al. demonstrated reduced liver fibrosis and inflammation under DPP4 inhibition by modulation of immune cells in a model of experimental liver injury, including a reduction in CD4 + lymphocytes^52,53^. These studies confirm our finding of an altered αβ T cell regulation with reduced cytokine production in the presence of DPP4 inhibition. However, as we did not analyze isolated αβ T cell cultures, we cannot definitively exclude contributions from other immune cell types—such as macrophages or monocytes—present in the mixed leukocyte cultures, which may have also contributed to the observed reduction in cytokine production. Examining the effect of DPP4 inhibition on isolated αβ T cells and their cytokine production would enable a more comprehensive assessment of the role of CD26/DPP4 in BA.

However, since IL-17 is exclusively produced by γδ T cells in the mouse model of BA^15^, the markedly reduced IL-17 levels observed following DPP4 inhibition in cultures of intrahepatic leukocytes from BA mice are likely attributable to decreased IL-17 production by γδ T cells. This is further supported by our findings in isolated γδ T cell cultures, where DPP4 inhibition similarly led to a reduction in cytokine production. To the best of our knowledge this is the first time an effect of DPP4 inhibition on cytokine production of γδ T cells has been demonstrated. Thus, comparable data at this point does not exist. Further experiments are needed to confirm our observations of DPP4 inhibition in this T cell subpopulation.

### Clinical effects of DPP4 Inhibition in the mouse model

In contrast to the highly significant reduction of pro-inflammatory cytokines after DPP4 inhibition in vitro, we observed rather mild clinical effects of Sitagliptin treatment in the mouse model of BA. The observed reductions in GOT and bilirubin levels in sitagliptin-treated animals are potentially attributable to decreased inflammatory activity and, consequently, amelioration of liver damage. Supporting this, a reduction in hepatic F4/80⁺ macrophages was also observed, indicating attenuated liver inflammation. However, GPT levels—which are considered more specific indicators of hepatocellular injury—were not significantly altered. In contrast, GOT, which is expressed in various tissues^54^, may reflect a broader, systemic response to DPP4 inhibition rather than a liver-specific effect. These findings provide proof that DPP4 inhibition can modulate inflammatory responses in experimental BA, but the effects appear to be non-specific and not uniquely confined to the hepatic environment. This supports our earlier conclusion that DPP4 plays a limited, non-specific role in BA.

The modest clinical effects observed in the mouse model may also be attributed to the incomplete DPP4 inhibition to about 50% residual activity, contrasting the nearly complete inhibition in vitro. We administered a dosage of 20 mg/kg per day of Sitagliptin, an estimate based on studies with adult mice where 10–100 mg/kg per day Sitagliptin were successfully used^55,56^. No data on neonatal mice was available and a further increase of dosage was not possible due to local animal welfare restrictions. Whether higher doses could elicit stronger clinical effects remains unclear. A dose-escalation study would have enabled a more comprehensive evaluation of sitagliptin’s efficacy; however, due to limited data on neonatal application, treatment was restricted to a single literature-based dose. Additionally, a longer follow-up of treated mice would have been desirable, but was challenging to implement given the acute progression of the BA mouse model.

Additional limitations of our study include the lack of detailed analyses of the signaling pathways underlying the observed decrease in cytokine production following DPP4 inhibition. Given the complexity of these pathways, further studies focusing on the molecular mechanisms involved are warranted to deepen our understanding. Moreover, our data do not allow us to distinguish between the enzymatic and co-stimulatory functions of CD26 that may contribute to the observed in vitro and in vivo effects. Future experiments using enzymatically inactive CD26 mutants or blocking antibodies could help clarify the mechanisms at play.

In conclusion, although CD26/DPP4 is upregulated in BA, this may reflect a general immune response rather than a unique involvement of CD26, which likely represents only one of many factors within the complex immunoregulatory network of BA. Whether CD26/DPP4 could play a meaningful role in the therapy of BA remains uncertain, given the limited clinical effects we observed. Further studies evaluating different DPP4 inhibitors and investigating their pharmacodynamic profiles in more detail are needed to determine whether DPP4 inhibition could meaningfully reduce hepatic inflammation in BA.

## Supplementary Information

Below is the link to the electronic supplementary material.

Supplementary Material 1

## Data Availability

All relevant data are included in the manuscript and supplementary files. Additional data are available from the corresponding authors upon reasonable request.
