# Supplementary material for: DPP4-inhibition reduces pro-inflammatory cytokine production by alpha-beta and gamma-delta T cells in vitro and in the biliary atresia mouse model
Source: Sci Rep. 2025 Aug 18;15:30226. doi: 10.1038/s41598-025-16097-z (PMC12361430; doi:10.1038/s41598-025-16097-z)
Supplement: Supplementary file 1 — Supplementary Material 1 [file 41598_2025_16097_MOESM1_ESM.docx]

**
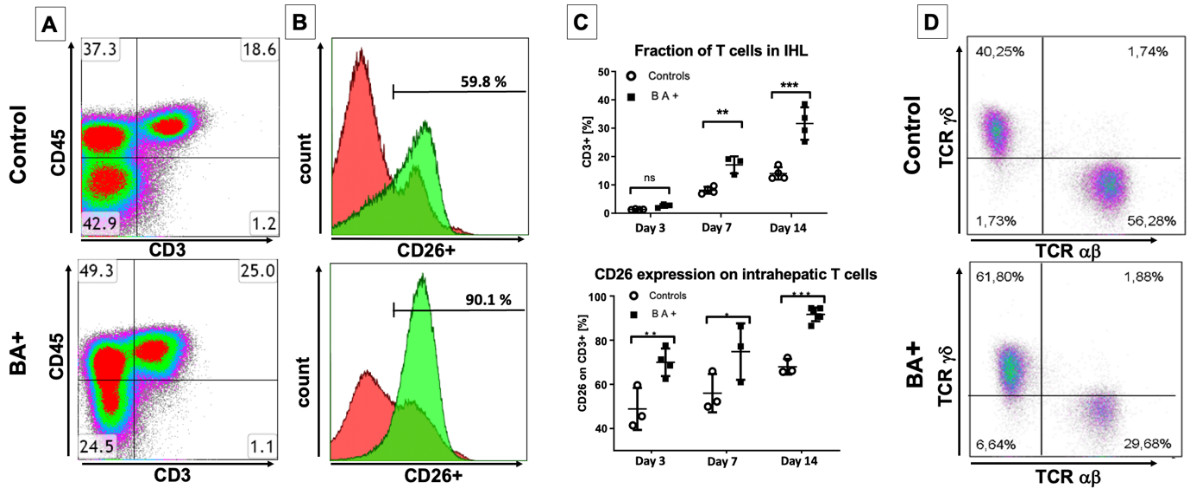
**

**Suppl. Figure 1: T cell fraction and their CD26 expression increases in intrahepatic leukocytes of mice with BA**. Balb/c mice were injected with PBS or RRV within 24 hours of life. Leukocytes were isolated from the livers of 6-12 mice on day of life (DOL) 3, 7, and 14. Cells were pooled from 2-3 mice for further analysis via flow cytometry. Flow cytometry analysis of T cells was performed using a panel targeting murine surface markers, including TCRγδ, CD26, CD3e, CD8a, TCRβ, CD4, and CD45. Cells were first stained with viability dye, followed by surface staining with specific antibodies. **A**: Viable mononuclear (MNC) CD45^+^ T cells were gated for CD3^+^/CD3^-^. The gating strategy is shown in Suppl. Fig. 2. **B/C**: CD26 expression was measured on CD3^+^/CD26^+^ cells from (A). **D**: γδ T cell fraction and αβ T cell fraction of liver infiltrating leucozytes on day 3 after induction of BA (Gate: viable MNC^+^CD45^+^CD3^+^ cells). Data represent three independent experiments. Error bars represent the standard deviation. Groups were compared using unpaired t-test. *p<0.05; **p<0.01; ***p<0.001; ns= not significant.


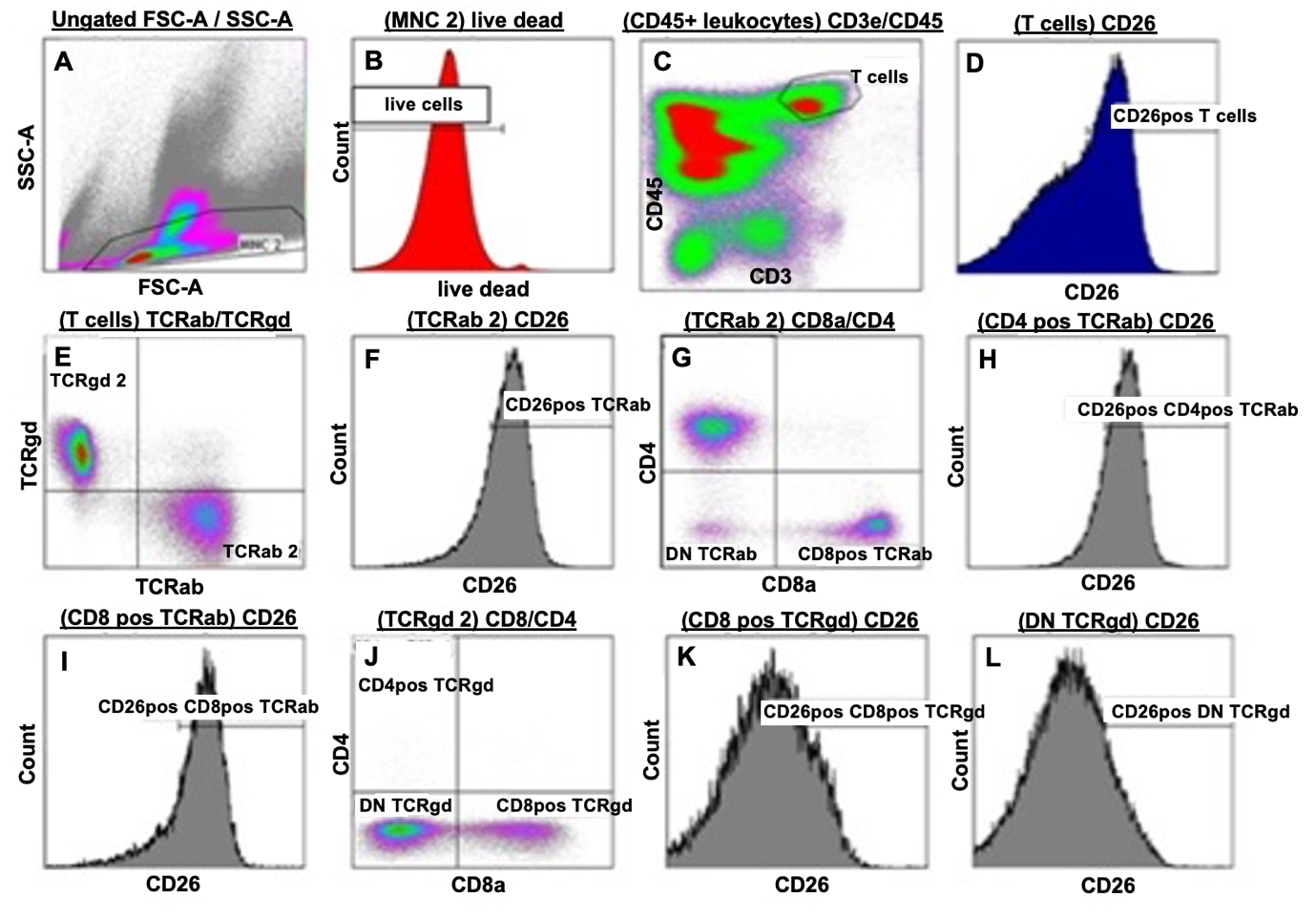


**Suppl. Figure 2: Gating strategy for FACS Analysis.**

Leucocytes were isolated from spleen and lymph nodes of healthy adult mice. A minimum of 5x 10^5^ cells was used per staining. If necessary, cells were pooled from 2-3 animals of the same group. All antibodies were acquired from eBioscience, Frankfurt, Germany, dilutions are specified in parentheses. **A:** Forward versus side scatter (FSC vs. SSC) gating of mononuclear cells. **B**: eF506 viability dye (1:1000) was used to exclude dead cells. **C:** T cells were gated for CD3^+^ and CD45^+^ using antibodies PerCP-Cy5.5 (1:200) and eF450 (1:600). **D:** Total CD26^+^ T cells were identified using PE antibody (1:50). **E:** Total T Cells were differentiated into γδ T cells (TCRgd 2) using antibody FITC (1:100) and αβ T cells (TCRab 2) using antibody APC (1:600). αβ T cells were further differentiated into CD26^+^αβ T cells with PE antibody (1:50) **(F)** as well as CD4^+^αβ T cells (antibody eF780, 1:600) and CD8^+^αβ T cells (PE-Cy7, 1:200) **(G)**. The fraction of CD26^+^ T cells of CD4^+^ αβ T cells and CD8^+^ αβ T cells is shown in **H** (CD3^+^/CD45^+^/CDab^+^/CD4^+^/CD26^+^) and **I** (CD3^+^/CD45^+^/CDab^+^/CD8^+^/CD26^+^), respectively.  **J:** gd T cells were differentiated into CD^-/-^ (DN TCRgd) and CD8^+^ gd T cells (CD3^+^/CD45^+^/TCRgd^+^/ CD4^-^/CD8^+^). CD26 expression was characterized on both subpopulations (**K** + **L**)**.**

**
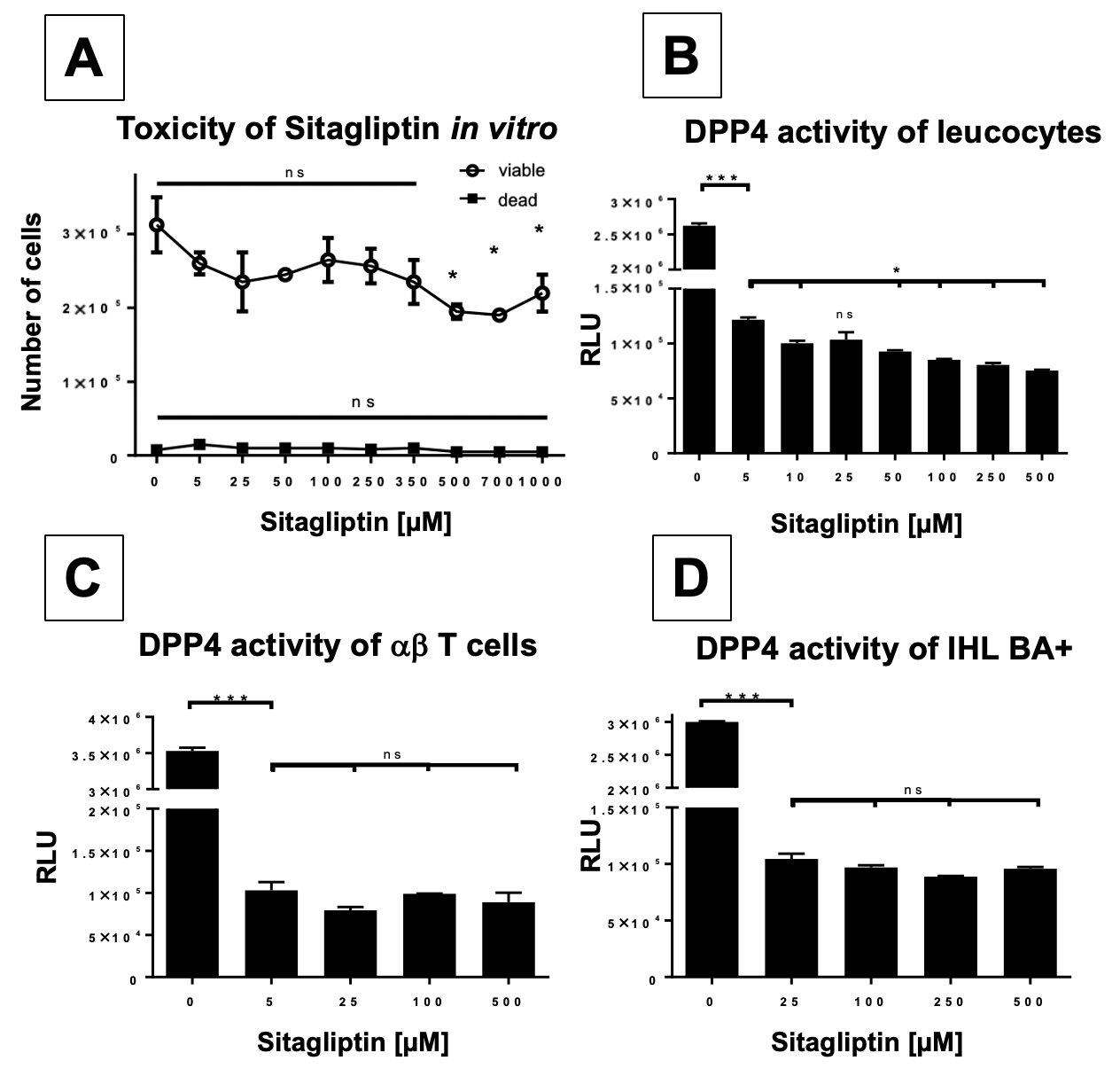
**

**Suppl. Figure 3: Sitagliptin inhibits DPP4 activity *in vitro*.**

**A**: Leukocytes isolated from the spleen and lymph nodes of healthy mice (n=2), plated out at a concentration of 3x10^5^ cells per well, were stimulated with soluble anti-CD3-antibodies and incubated with increasing concentrations of Sitagliptin for 72 hours. Viable and dead cells were counted using trypan blue dye exclusion test. **B**: Supernatants from (A) were analyzed for DPP4 activity using a bioluminescence assay. **C**: αβ T cells were isolated from the spleen and lymph nodes of healthy mice (n=4) by MACS. 2x10^5^ cells per well were stimulated with soluble anti-CD3-antibodies and co-incubated with increasing concentrations of Sitaglipin in triplicates for 72 hours. DPP4 activity in supernatants was measured. **D**: The same procedure as in (C) was applied to intrahepatic leukocytes from mice with BA (n=6) taken on Day of life (DOL) 14.

Error bars represent the standard deviation. Unpaired t-test was used for comparison of two groups and ANOVA for analysis of more than two groups. * p<0.05; *** p<0.001; ns = not significant. (RLU = relative light units, IHL = intrahepatic leucocytes).
